# Effects of undenatured whey protein supplementation on CXCL12- and CCL21-mediated B and T cell chemotaxis in diabetic mice

**DOI:** 10.1186/1476-511X-10-203

**Published:** 2011-11-09

**Authors:** Gamal Badr, Mohamed Mohany, Ali Metwalli

**Affiliations:** 1 Zoology Department, College of Science, King Saud University, Saudi Arabia; 2 Zoology Department, Faculty of Science, Assiut University, Egypt; 3 Food Science Department, College of Agriculture and Food Science, King Saud University, Saudi Arabia

**Keywords:** B cells, chemotaxis, diabetes mellitus, F-actin polymerization, T cells, whey protein

## Abstract

**Background:**

Long and persistent uncontrolled diabetes tends to degenerate the immune system and leads to an increased incidence of infection. Whey proteins (WPs) enhance immunity during early life and have a protective role in some immune disorders. In this study, the effects of camel WP on the chemotaxis of B and T cells to CXCL12 and CCL21 in diabetic mice were investigated.

**Results:**

Flow cytometric analysis of the surface expressions of CXCR4 (CXCL12 receptor) and CCR7 (CCL21 receptor) on B and T cells revealed that the surface expressions of CXCR4 and CCR7 were not significantly altered in diabetic and WP-supplemented diabetic mice compared with control mice. Nevertheless, B and T lymphocytes from diabetic mice were found to be in a stunned state, with a marked and significant (*P *< 0.05) decrease in CXCL12- and CCL21-mediated actin polymerization and subsequently, a marked decrease in their chemotaxis. WP supplementation in the diabetes model was found to significantly increase CXCL12- and CCL21-mediated actin polymerization and chemotaxis in both B and T cells.

**Conclusion:**

Our data revealed the benefits of WP supplementation in enhancing cytoskeletal rearrangement and chemotaxis in B and T cells, and subsequently improving the immune response in diabetic mice.

## Background

Type 1 diabetes is defined as a complex multifactorial disease in which genetic factors with environmental modifiers give rise to immune abnormalities, leading to pancreatic β-cell damage and destruction. Diabetes mellitus is usually associated with many metabolic complications [1]. In diabetic patients, infections occur with greater frequency and severity than in non-diabetics due to both humoral and cellular immune response impairment [2]. Numerous defects have been identified in CD8+ CD28 T-suppressor lymphocyte populations in patients with type 1 diabetes mellitus and multiple sclerosis [3]. Some evidence has suggested that defects in immune cells might interfere with normal pancreatic development and glucose homeostasis [4]. Additionally, a recent study reported that diabetic patients have demonstrable defects in lymphocyte function due to disruptions in potassium channels [5]. A previous investigation demonstrated that monocytes isolated from diabetic patients spontaneously secreted proinflammatory cytokines, leading to an altered T cell response [6]. Secondary lymphoid tissues are sites of antigen recognition in which B and T cells associate with antigen-presenting cells (APCs) to initiate an adaptive immune response [7]. Chemokines play a crucial role in immune cell chemotaxis. In particular, CCL21 participates in naive T and B cell recruitment to the extra-follicular area in secondary lymphoid organs [8]. These chemokines, including CCL21 and CXCL12, are produced by cells scattered throughout the extra-follicular area and act through CXCR4 and CCR7, respectively, which are specifically expressed on activated T and B cells [9]. During B and T cell chemotaxis, the actin cytoskeleton is dynamically remodeled; this reorganization produces the force necessary for the activation and migration of these cells [10].

Whey proteins (WPs) represent a heterogeneous group of proteins (i.e., β-lactoglobulin, α-lactalbumin, serum albumin, and immunoglobulins). Indeed, recently published data have suggested that WP has antioxidant activity, likely due to cysteine abundance or the presence of glutamylcysteine groups, which are also found in other food proteins. Therefore, WP may be a therapeutic tool for oxidative stress-associated diseases [11]. Previous studies have reported that WPs and peptides derived from the enzymatic proteolysis of WPs modulate a variety of immune functions, including lymphocyte activation and proliferation, cytokine secretion, antibody production, phagocytic activity, and granulocyte and natural killer (NK) cell activity [12]. Another study revealed that oral administration of an undenatured cysteine-rich WP isolate increased glutathione (GSH) levels in several glutathione-deficient patient groups, including patients with advanced human immunodeficiency virus (HIV) [13]. In addition, *in vitro *and *in vivo *studies have demonstrated a clear modulation of immune functions by several whey protein-derived products [14]. Furthermore, whey peptides possess immunomodulatory activities, such as stimulating lymphocytes and increasing phagocytosis, and the secretion of immunoglobulin A (IgA) from Peyer's patches [15]. Recently, it has been reported that WP has immunomodulatory properties and the potential to increase host defense [16], protective effects against childhood asthma and atopic syndrome [17], and anticancer effects [18].

To further elucidate the potential benefits of this protein, this study investigated the impact of dietary supplementation with a camel WP on the actin polymerization and chemotaxis of B and T cells in response to CXCL12 and CCL21 in diabetic mice.

## Results

### WP supplementation does not affect the surface expression of CXCR4 and CCR7 on B and T cells in diabetic mice

**Figure 1:**
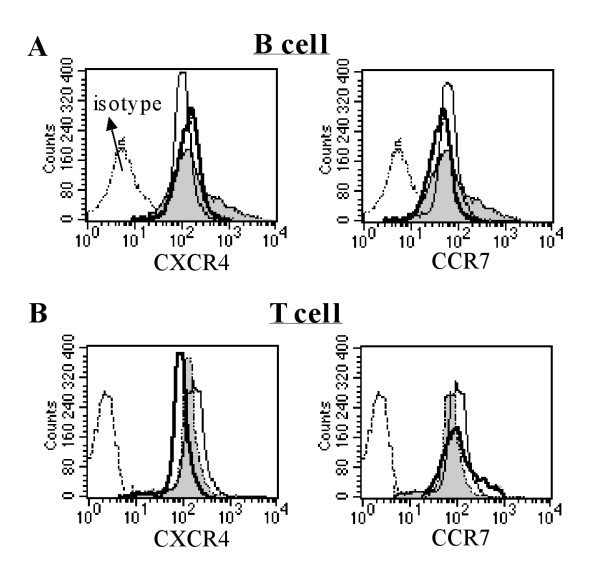
**Surface expression of CXCR4 and CCR7 on B and T cells**. Surface CXCR4 and CCR7 expression levels were analyzed with flow cytometry on B and T cells of the control mice (open histograms, thin line), diabetic mice (open histograms, black bold line) and diabetic mice supplemented with WP (gray-filled histograms, thin line) using anti-CXCR4-PE, anti-CCR7-PE and IgG (open histogram) isotype control antibodies. Histograms were gated based on viable B220+ B cells (A) and CD3+ T cells (B).

First, we analyzed the surface expressions of CXCR4 and CCR7 by flow cytometry on B and T cells in control mice, diabetic mice and diabetic mice supplemented with WP using anti-CXCR4-PE, anti-CCR7-PE and IgG isotype control antibodies. Histograms were gated based on viable B220+ B cells (Figure 1A) and CD3+ T cells (Figure 1B). We observed a slight but not significant decrease in the surface expression of chemokine receptors CXCR4 and CCR7 on both B and T cells in diabetic mice compared with control mice. Additionally, WP supplementation in the diabetes model had no significant effects on the surface expressions of these receptors.

**Figure 2:**
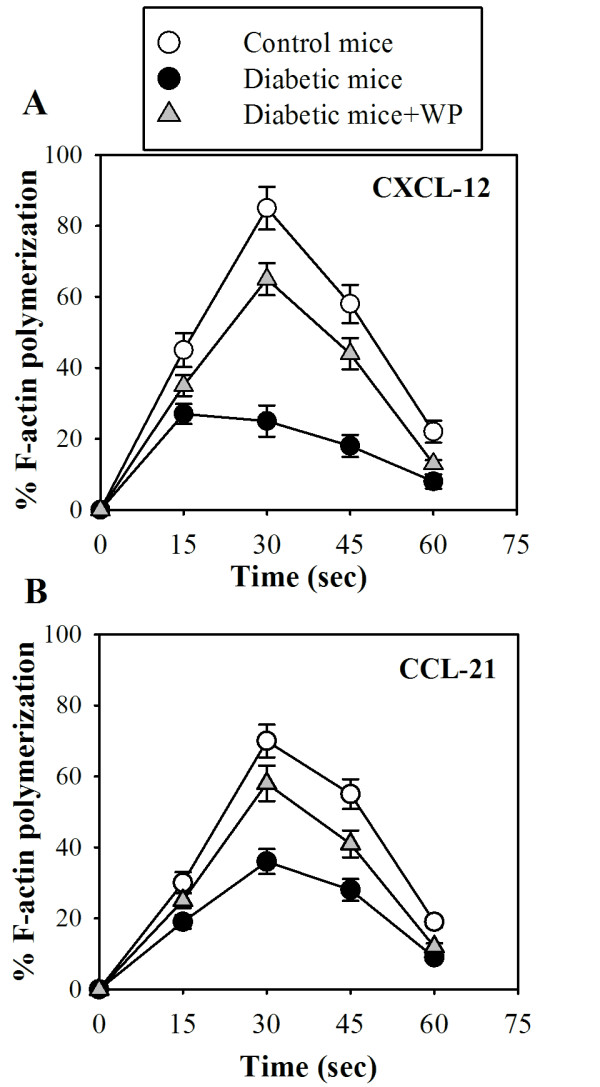
**Impact of WP supplementation on actin polymerization in B cells**. B cells were isolated from control (open circles), diabetic (closed black circles), and WP-treated diabetic groups (closed gray triangles). F-actin polymerization was measured in response to CXCL12 (A) and CCL21 (B). The results are expressed as the percentage changes in MFI (*n *= 10) ± SD as described in the Materials and Methods section.

### Whey protein supplementation reverses the impairment of chemokine-mediated actin polymerization in B cells in diabetic mice

Actin and microtubules provide the dynamic cellular framework required to orchestrate and ultimately control cellular activation, immunological synapse formation, proliferation and chemotaxis. Therefore, chemokine-mediated actin polymerization was monitored in B cells in the three animal groups. F-actin polymerization was measured in response to CXCL12 (Figure 2A) and CCL21 (Figure 2B) in B cells isolated from control (open circles), diabetic (closed black circles), and WP-treated diabetic mice (closed gray triangles). Fixed cells were analyzed by flow cytometry, and the mean fluorescence intensity (MFI) was determined for each sample. We found that the percentage of F-actin polymerization was significantly reduced in diabetic mice compared with the control group upon stimulation with CXCL12 or CCL21 every 15 seconds (*P *< 0.05; *n *= 10). In contrast, WP-treated diabetic mice exhibited a significant increase in chemokine-mediated actin polymerization.

### Whey protein supplementation reverses impairment of chemokine-mediated F-actin polymerization in T cells in diabetic mice

**Figure 3:**
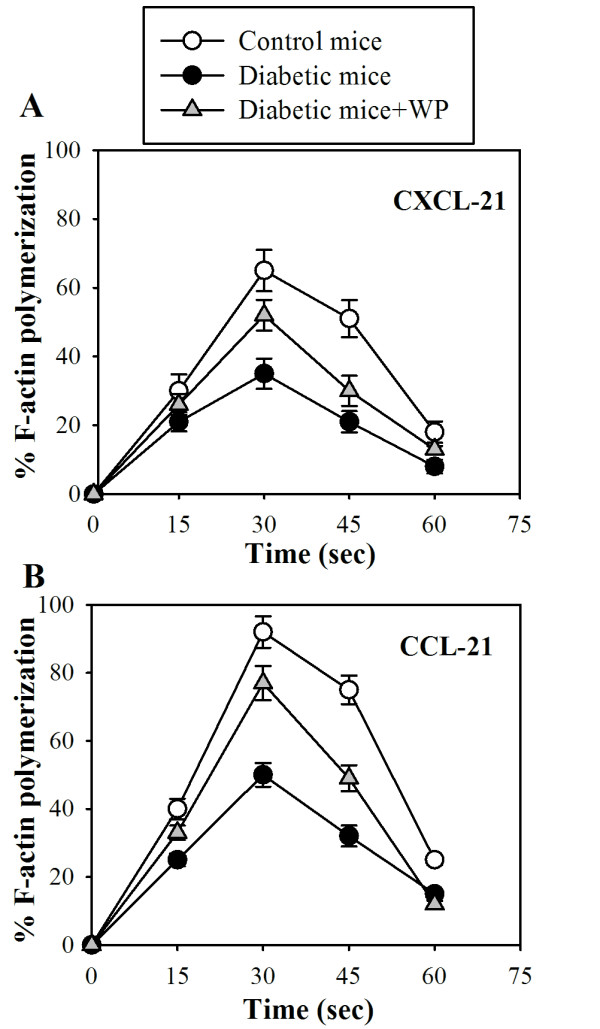
**Impact of WP supplementation on actin polymerization in T cells**. T cells were isolated from the control (open circles), diabetic (closed black circles), and WP-treated diabetic groups (closed gray triangles). F-actin polymerization was measured in response to CXCL12 (A) and CCL21 (B). The results are expressed as the percentage changes in MFI (*n *= 8) ± SD as described in the Materials and Methods section.

As mentioned previously, actin and microtubules provide the dynamic cellular framework to orchestrate and ultimately control T cell activation. Therefore, chemokine-mediated actin polymerization was also monitored in T cells in the three animal groups. The degree of F-actin polymerization was determined by flow cytometry in response to CXCL12 (Figure 3A) and CCL21 (Figure 3B). We observed that the percentage of F-actin polymerization was significantly reduced in diabetic mice (closed black circles) compared with the control mice (open circles) upon stimulation with CXCL12 or CCL21 every 15 seconds (*P *< 0.05; *n *= 8). In contrast, WP supplementation was found to partially restore the impairment of chemokine-induced F-actin polymerization in T cells in diabetic mice.

### Supplementation with WP improves and partially restores B cell chemotaxis in diabetic mice

**Figure 4:**
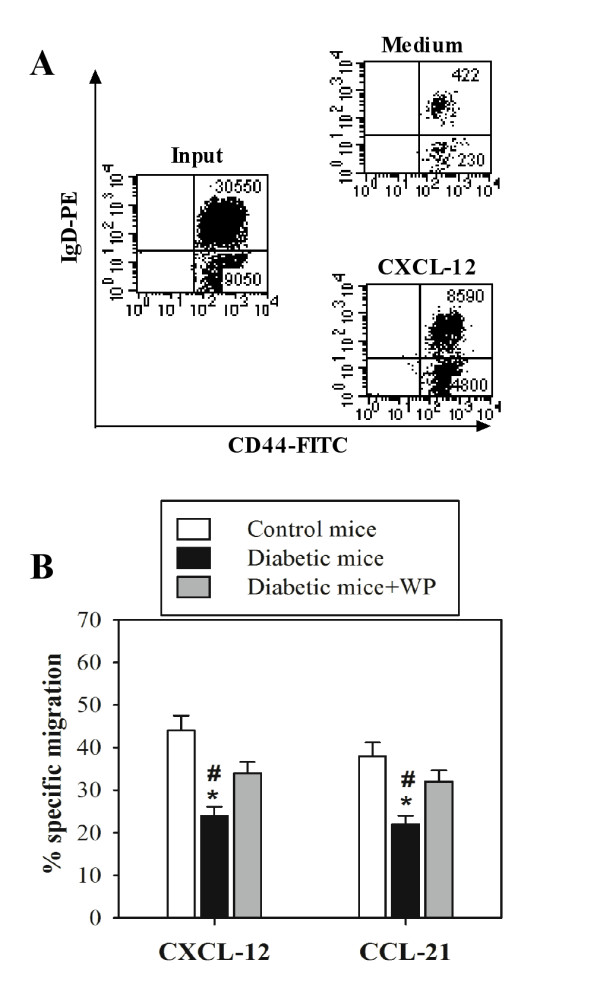
**Whey protein restores B cell chemotaxis in diabetic mice**. Splenic B cells were isolated from 10 mice in each group and were analyzed for migration to CXCL12 and CCL21. Input populations and migrated cell populations were stained with B220-APC and IgD-PE/CD44-FITC. Representative dot plots of input cells and transmigrated cells to medium (without chemokine) versus medium containing CXCL12 for control mice are shown, including the input and transmigrated B cell population data. Data from one representative experiment are shown in panel A. The percentage of B cells from 10 different mice in the control (open bars), diabetic (closed, black bars) and WP-treated diabetic (closed, gray bars) groups that specifically migrated to chemokines are shown. The results are expressed as the mean specific migration ± SEM. *P < 0.05, diabetic vs. control; **^#^***P *< 0.05, diabetic vs. WP-treated groups.

We assessed the chemotactic response of B cells isolated from the spleens of 10 mice from each group to CXCL12 and CCL21. Input and migrated cell populations were stained with B220-APC, IgD-PE and CD44-FITC. Dot plots of input cells and transmigrated cells to medium (without chemokine) versus medium containing CXCL12 for control mice are shown in Figure 4A (one representative experiment is shown) with the input and transmigrated B cell population data. Additionally, the percentages of B cell chemotaxis to CXCL12 and CCL21 from the control (open bars), diabetic (closed, black bars) and WP-treated diabetic (closed, gray bars) groups that specifically migrated to chemokines are shown in Figure 4B. These data revealed that the percentage of B cell chemotaxis was significantly decreased in diabetic mice compared with the control group (*P < 0.05), whereas WP-treated diabetic mice exhibited a significant increase in B cell chemotaxis compared with untreated diabetic mice (**^#^***P *< 0.05).

### WP supplementation improves T cell chemotaxis in diabetic mice

**Figure 5:**
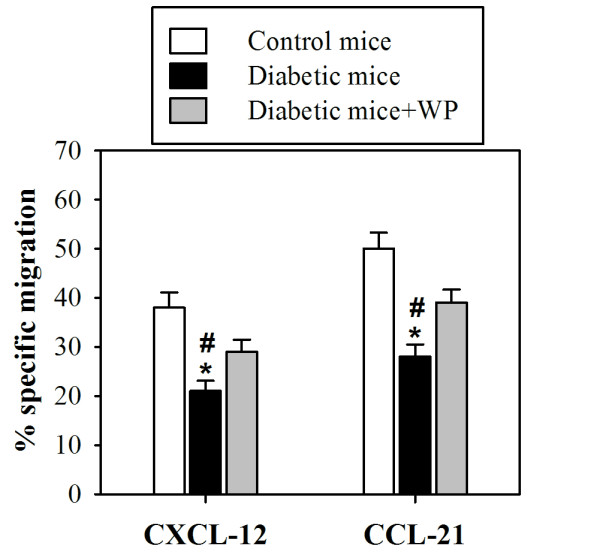
**Whey protein restores T cell chemotaxis in diabetic mice**. T cells were isolated from 10 mice in each group and analyzed for migration to CXCL12 and CCL21. Input populations and migrated cell populations were stained with CD3-FITC. The percentage of T cells from 10 different mice in the control (open bars), diabetic (closed, black bars) and WP-treated diabetic (closed, gray bars) groups that specifically migrated to chemokines are shown. The results are expressed as the mean specific migration ± SEM. *P < 0.05, diabetic vs. control; **^#^***P *< 0.05, diabetic vs. WP-treated groups.

The percentage of T cells isolated from the spleens and peripheral lymph nodes (PLNs) that specifically migrated were assessed with flow cytometry. Input populations and migrated cell populations were stained with CD3-FITC. The percentages of T cells in control (open bars), diabetic (closed, black bars) and WP-treated diabetic (closed, gray bars) groups that specifically migrated to chemokines are shown **(**Figure 5**)**. Our data demonstrated that the percentage of T cell chemotaxis was significantly decreased in diabetic mice compared with control mice (*P < 0.05; *n *= 10), and WP-treated diabetic mice exhibited a significant increase in T cell chemotaxis compared with untreated diabetic mice (**^#^***P *< 0.05; *n *= 10).

## Discussion

Antioxidants play a vital role in maintaining immunity and protection against free radical damage in the human body. Whey proteins have utility in managing many different diseases, including fatty liver disease [19], cardiovascular diseases [20], colitis [21], HIV infection [13], and cancer [18]. CXCL12 and CCL21, via their cognate receptors (CXCR4 and CCR7, respectively), play a central role in lymphocyte trafficking and homing to secondary lymphoid organs, where they recognize presented antigens. CCR7 and CXCR4 are expressed on all naive T cells, some memory T cells, B cells, and mature dendritic cells and play a central role in lymphocyte trafficking and homing to lymph nodes. We first monitored the surface expression of CXCR4 and CCR7 with flow cytometry on B and T cells in control mice, untreated diabetic mice and diabetic mice supplemented with WP. We observed that there were no significant differences in the surface expression of CXCR4 and CCR7 in both B and T cells in all groups throughout the experimental period. Although WP supplementation did not affect the surface expression of CXCR4 and CCR7 on B and T cells, the activity and underlying signaling proteins of these receptors might be altered; this requires further study. Actin polymerization plays a critical role in lymphocyte activation and subsequent signaling, cell migration, morphogenesis, cellular trafficking, cytokinesis and cell proliferation [22]. In this study, it was determined that WP supplementation reversed chemokine-mediated actin polymerization impairment in both B and T cells in diabetic mice. Similar observations have been made by [23], who observed actin polymerization impairment in type 2 diabetes. To our knowledge, we show for the first time that WP increased chemokine-mediated actin polymerization in these lymphocytes. Defective actin polymerization leads to long-term T cell unresponsiveness or anergy [24]. Our results are in agreement with the results of [25], who found that vitamin C sustains TCR-mediated actin polymerization and T cell responsiveness in streptozotocin-induced diabetic rats. Chemotaxis is an essential phenomenon for shaping immune responses, and chemokine-receptor antagonists are now being evaluated as therapies for various inflammatory and autoimmune diseases. In this study, these data revealed that the percentage of chemotactic B cells was significantly impaired in diabetic mice, whereas WP-treated diabetic groups exhibited significant increases in chemotactic B and T cells. A previous study reported that CD34+ cells isolated from diabetic patients demonstrated a marked defect in migration to SDF-1 (i.e., CXCL12), supporting our results [26]. Moreover, previous investigations have shown that the CXCR4/CXCL12 (SDF-1) signaling pathway protects non-obese diabetic mice from autoimmune diabetes [27]. Our results showed that WP increased CCL21- and CXCL12-mediated B and T cells chemotaxis in diabetic mice, which is considered a unique and major finding. Previous studies have shown that CCR7 and CXCR4 are involved in the recruitment of blood-borne leukocytes to sites of inflammation [28]. As receptors, CXCR4 and CCR7 can be considered activation markers [29]. Recently, reports have shown that bovine WP enhances innate immunity by increasing neutrophil chemotaxis [30]. Our data suggest improved B and T cell chemotaxis efficiency following WP treatment in diabetic mice.

## Conclusions

In conclusion, these data expand our knowledge of WP supplementation in enhancing the immune response in diabetic mice by increasing chemokine-mediated actin polymerization and chemotaxis in both B and T cells, suggesting that WP may be a promising drug candidate for immunomodulation.

## Materials and methods

### Preparation of whey proteins

Raw camel milk was collected from healthy female camels (three camel breeds: Majaheim, Maghateer, and Soffer) from the Riyadh area in Saudi Arabia. The milk was then centrifuged to remove the cream. The skim milk obtained was acidified to pH 4.3 using 1 N HCl at room temperature and centrifuged at 10,000 × *g *for 10 min to precipitate the casein. The resultant whey, which contained the whey proteins, was saturated with ammonium sulfate to a final saturation of 80% to precipitate the whey proteins. The precipitated whey proteins were dialyzed against 20 volumes of distilled water for 48 hr using a molecular-porous membrane with an Molecular Weight Cut Off (MWCO) of 6,000-8,000 kDa. The dialysate containing undenatured whey proteins was freeze-dried and refrigerated until use.

### Chemicals

Streptozotocin (STZ) was obtained from Sigma Chemicals Co., St. Louis, MO, USA. STZ was dissolved in cold 0.01 M citrate buffer (pH 4.50) and was always freshly prepared for immediate use (within 5 min).

### Animals and experimental design

A total of 30 sexually mature 12-week-old male mice weighing 25-30 g each were obtained from the Central Animal House of the Faculty of Pharmacy at King Saud University. All animal procedures were conducted in accordance with the standards set forth in the Guidelines for the Care and Use of Experimental Animals by the Committee for the Purpose of Control and Supervision of Experiments on Animals (CPCSEA) and the National Institutes of Health (NIH). The study protocol was approved by the Animal Ethics Committee of the Zoology Department, College of Science, King Saud University according to the Helsinki principles. All animals were allowed to acclimate to the metal cages inside a well-ventilated room for 2 weeks prior to experimentation. Animals were maintained under standard laboratory conditions (temperature 23°C, relative humidity 60-70% and a 12-hour light/dark cycle), fed a diet of standard commercial pellets and given water ad libitum. All mice were fasted for 20 hr before diabetes induction. Mice (*n *= 20) were rendered diabetic with an intraperitoneal injection (i.p.) of a single dose of STZ (60 mg/kg body weight) in 0.01 M citrate buffer (pH 4.5) [31]. Mice in the control group (*n *= 10) were injected with the vehicle alone (0.01 M citrate buffer, pH 4.5). The animals were divided into three experimental groups: group 1, control non-diabetic mice; group 2, diabetic mice; and group 3, diabetic mice orally supplemented with undenatured WP (100 mg/kg body weight/day for one month through oral gavage).

### Total lymphocyte and B and T cell isolation

Lymphocytes were isolated from the spleens and PLNs of adult male mice. Lymph nodes and spleens were homogenized using 40-μm cell strainers (BD Falcon, Bedford, MA, USA). Red blood cells from spleens were osmotically lysed using ACK buffer. Cells were washed with phosphate-buffered saline (PBS), counted using the trypan blue exclusion test, and cultured in R-10 culture medium (complete RPMI 1640 medium supplemented with 10% FCS, 2 mM L-glutamine, 100 IU/ml penicillin, 100 μg/ml streptomycin, 1 mM sodium pyruvate, and 50 μM 2-mercaptoethanol).

Splenic B cells were isolated by negative depletion by using biotinylated antibodies against CD4, CD8, GR-1, and CDllc and Dynabeads M-280 Streptavidin (Invitrogen, France) as previously described [32]. Splenic T cells were also isolated using negative selection columns. Cell purity was assessed with flow cytometry and was greater than 95%. Cells were cultured in R-10 medium.

### Antibodies and flow cytometry

Lymphocytes from PLNs, spleens, blood and purified cell populations (1 × 10^6 ^cells per 50 μl PBS) were blocked with purified CD16/CD32 FccII/III mAb to prevent non-specific binding. Subsequently, cells were stained with mAbs and analyzed using a FACSCalibur flow cytometer (BD, Franklin Lakes, NJ, USA). Antibodies against mouse CD11b, CD11c, CD3, CD4, CD8a, and B220 were purchased from BD Pharmingen. Anti-mouse CCR7, CXCR4, CCL21 and CXCL12 were purchased from R&D Systems.

### F-actin polymerization assay

Intracellular F-actin polymerization was assessed as previously described [9]. Cells from each group (8 × 10^6^/ml) were incubated in HEPES-buffered RPMI 1640 at 37°C and stimulated with or without chemokines. At the indicated times, cells (100 μl) were added to 400 μl of assay buffer containing 4 × 10^-7 ^M FITC-labeled phalloidin, 0.5 mg/ml L-α- lysophosphatidylcholine (both from Sigma-Aldrich) and 4.5% formaldehyde in PBS. Fixed cells were analyzed with flow cytometry, and the mean fluorescence intensity (MFI) was determined for each sample. The percent change in MFI was calculated for each sample at each time point as follows: [1-(MFI before addition of chemokine/MFI and after addition of chemokine)] × 100.

### In vitro chemotaxis assay

The chemotaxis of B and T cells was measured using migration through polycarbonate filters with a 3-μm pore size in 24-well transwell chambers (Corning Costar, Cambridge, MA, USA) as previously described [25]. Briefly, 1 × 10^5 ^B or T cells or DCs in 100 μl of pre-warmed migration medium (RPMI 1640 containing 10 mM HEPES and 1% FBS) were added to the upper chamber. A total of 600 μl of migration medium containing 250 ng/ml CCL21 or CXCL12 or medium alone as a control for spontaneous migration was added to the lower chamber, and the cells were incubated for 3 hours at 37°C. Cells that transmigrated to the bottom chamber were collected and counted with flow cytometry (FACSCalibur, BD, Franklin Lakes, NJ) for a fixed period of 60 seconds. The mean number of spontaneously migrated cells was subtracted from the total number of migrated cells. The results are shown as the percentage of specific migration ± SEM.

### Statistical analysis

Data are expressed as the mean ± SEM (standard error of the mean). Significant differences between groups were analyzed with a one-way analysis of variance (for more than two groups) followed by Tukey's post-test using SPSS software, version 17. Differences were considered statistically significant at *P *< 0.05.

## Competing interests

The authors declare that they have no competing interests.

## Authors' contributions

GB put the design of the study, carried out the immunological assays, prepared figures, drafted the manuscript and performed the statistical analysis. MM was responsible of the animal model and participated in drafting the manuscript. AM was responsible for the extraction and preparation of the Whey protein. All authors read and approved the final manuscript.

## References

[B1] Thompson CS Animal models of diabetes mellitus: relevance to vascular complications Curr Pharm Des 2008 14 309 324 10.2174/138161208783497679 18289058

[B2] Smitherman KO Peacock JE Infectious emergencies in patients with diabetes mellitus Med Clin North Am 1995 79 53 77 7808095 10.1016/s0025-7125(16)30084-0

[B3] Mikulkova Z Praksova P Stourac P Bednarik J Strajtova L Pacasova R Belobradkova J Dite P Michalek J Numerical defects in CD8+CD28- T-suppressor lymphocyte population in patients with type 1 diabetes mellitus and multiple sclerosis Cell Immunol 2010 262 75 9 10.1016/j.cellimm.2010.02.002 20219185

[B4] Zeidler A Shargill NS Chan TM Peripheral insulin insensitivity in the hyperglycemic athymic nude mouse: similarity to noninsulin-dependent diabetes mellitus Proc Soc Exp Biol Med 1991 196 457 460 2008443 10.3181/00379727-196-43216

[B5] Toldi G Vásárhelyi B Kaposi A Mészáros G Pánczél P Hosszufalusi N Tulassay T Treszl A Lymphocyte activation in type 1 diabetes mellitus: the increased significance of Kv1.3 potassium channels Immunol Lett 2010 133 35 41 10.1016/j.imlet.2010.06.009 20603149

[B6] Bradshaw EM Raddassi K Elyaman W Orban T Gottlieb PA Kent SC Hafler DA Monocytes from patients with type 1 diabetes spontaneously secrete proinflammatory cytokines inducing Th17 cells J Immunol 2009 183 4432 9 10.4049/jimmunol.0900576 19748982 PMC2770506

[B7] Von Andrian U Mackay C T-cell function and migration: two sides of the same coin N Engl J Med 2000 343 1020 1034 10.1056/NEJM200010053431407 11018170

[B8] Gunn MD Kyuwa S Tam C Kakiuchi T Matsuzawa A Williams LT Nakano H Mice lacking expression of secondary lymphoid organ chemokine have defects in lymphocyte homing and dendritic cell localization J Exp Med 1999 189 451 460 10.1084/jem.189.3.451 9927507 PMC2192914

[B9] Badr G Borhis G Treton D Richard Y IFN-alpha enhances human B-cell chemotaxis by modulating ligand-induced chemokine receptor signaling and internalization Int Immunol 2005 17 459 67 10.1093/intimm/dxh227 15749730

[B10] Pollard TD Borisy GG Cellular motility driven by assembly and disassembly of actin filaments Cell 2003 112 453 65 10.1016/S0092-8674(03)00120-X 12600310

[B11] Balbis E Patriarca S Furfaro A Millanta S Sukkar GS Marinari MU Pronzato AM Cottalasso D Traverso N Whey proteins influence hepatic glutathione after CCl4 intoxication Toxico Ind Heal 2009 25 325 328 10.1177/0748233709104870 19651804

[B12] Gauthier SF Pouliot Y Saint-Sauveur D Imunomodulatory peptides obtained by the enzymatic hydrolysis of whey proteins Inter Dairy J 2006 16 1315 1323 10.1016/j.idairyj.2006.06.014

[B13] Micke P Beeh KM Schlaak JF Buhl R Oral supplementation with whey proteins increases plasma glutathione levels of HIV-infected patients Eur J Clin Invest 2001 31 171 8 10.1046/j.1365-2362.2001.00781.x 11168457

[B14] Krissansen GW Emerging health properties of whey proteins and their clinical implications J Am Coll Nutr 2007 26 S713 23 10.1080/07315724.2007.10719652 18187438

[B15] Beaulieu J Dupont C Lemieux P Whey proteins and peptides: beneficial effects on immune health Therapy 2006 3 1 10 10.2217/14750708.3.1.1

[B16] Rusu D Drouin R Pouliot Y Gauthier S Poubelle PE A bovine whey protein extract stimulates human neutrophils to generate bioactive IL-1Ra through a NF-kappaB- and MAPK-dependent mechanism J Nutr 2010 140 2 382 91 10.3945/jn.109.109645 20032479

[B17] Loss G Apprich S Waser M Kneifel W Genuneit J Büchele G Weber J Sozanska B Danielewicz H Horak E van Neerven RJ Heederik D Lorenzen PC von Mutius E Braun-Fahrländer C GABRIELA study group. The protective effect of farm milk consumption on childhood asthma and atopy: The GABRIELA study J Allergy Clin Immunol 2011 128 766 773 10.1016/j.jaci.2011.07.048 21875744

[B18] Castro GA Maria DA Bouhallab S Sgarbieri VC *In vitro *impact of a whey protein isolate (WPI) and collagen hydrolysates (CHs) on B16F10 melanoma cells proliferation J Dermatol Sci 2009 56 51 7 10.1016/j.jdermsci.2009.06.016 19695839

[B19] Hamad EM Taha SH Abou Dawood AG Sitohy MZ Abdel-Hamid M Protective effect of whey proteins against nonalcoholic fatty liver in rats Lipids Health Dis 2011 10 57 10.1186/1476-511X-10-57 21489294 PMC3096574

[B20] Pal S Ellis V Ho S Acute effects of whey protein isolate on cardiovascular risk factors in overweight, post-menopausal women Atherosclerosis 2010 212 339 44 10.1016/j.atherosclerosis.2010.05.032 20561625

[B21] Sprong RC Schonewille AJ van der Meer R Dietary cheese whey protein protects rats against mild dextran sulfate sodium-induced colitis: role of mucin and microbiota J Dairy Sci 2010 93 1364 71 10.3168/jds.2009-2397 20338413

[B22] Uetrecht AC Bear JE Coronins: the return of the crown Trends Cell Biol 2006 16 421 426 10.1016/j.tcb.2006.06.002 16806932

[B23] Advani A Marshall SM Thomas TH Increasing neutrophil F-actin corrects CD11b exposure in Type 2 diabetes Eur J Clin Invest 2004 34 358 64 10.1111/j.1365-2362.2004.01346.x 15147333

[B24] Gomez TS Hamann MJ McCarney S Savoy DN Lubking CM Heldebrant MP Labno CM McKean DJ McNiven MA Burkhardt JK Billadeau DD Dynamin 2 regulates T cell activation by controlling actin polymerization at the immunological synapse Nat Immunol 2005 6 261 270 10.1038/ni1168 15696170

[B25] Badr G Sayed D Alhazza IM Elsayh KI Ahmed EA Alwasel SH T lymphocytes from malnourished infants are short-lived and dysfunctional cells Immunobiology 2011 216 309 15 10.1016/j.imbio.2010.07.007 20822829

[B26] Segal MS Shah R Afzal A Perrault CM Chang K Schuler A Beem E Shaw LC Li Calzi S Harrison JK Tran-Son-Tay R Grant MB Nitric oxide cytoskeletal-induced alterations reverse the endothelial progenitor cell migratory defect associated with diabetes Diabetes 2006 55 102 9 10.2337/diabetes.55.01.06.db05-0803 16380482

[B27] Aboumrad E Madec AM Thivolet C The CXCR4/CXCL12 (SDF-1) signalling pathway protects non-obese diabetic mouse from autoimmune diabetes Clin Exp Immunol 2007 148 432 9 10.1111/j.1365-2249.2007.03370.x 17374136 PMC1941939

[B28] Blades MC Manzo A Ingegnoli F Taylor PR Panayi GS Irjala H Jalkanen S Haskard DO Perretti M Pitzalis C Stromal cell-derived factor 1 (CXCL12) induces human cell migration into human lymph nodes transplanted into SCID mice J Immunol 2002 168 4308 11970972 10.4049/jimmunol.168.9.4308

[B29] Lastovicka J Budinsky V Spisek R Bartunková J Assessment of lymphocyte proliferation: CFSE kills dividing cells and modulates expression of activation markers Cell Immunol 2009 256 79 85 10.1016/j.cellimm.2009.01.007 19233349

[B30] Rusu D Drouin R Pouliot Y Gauthier S Poubelle PE A bovine whey protein extract can enhance innate immunity by priming normal human blood neutrophils J Nutr 2009 139 386 93 19106313 10.3945/jn.108.098459

[B31] Zhao J Weiler HA Long-term effects of gestational diabetes on offspring health are more pronounced in skeletal growth than body composition and glucose tolerance Br J Nutr 2010 9 1 9 10.1017/S0007114510002631 20615268

[B32] Moratz C Hayman JR Gu H Kehrl JH Abnormal B-cell responses to chemokines, disturbed plasma cell localization, and distorted immune tissue architecture in Rgs1-/- mice Mol Cell Biol 2004 24 5767 75 10.1128/MCB.24.13.5767-5775.2004 15199133 PMC480912

